# Editorial: Forest insect invasions – risk mapping approaches and applications

**DOI:** 10.3389/finsc.2024.1378061

**Published:** 2024-03-18

**Authors:** Kishan R. Sambaraju, Vivek Srivastava, Brittany S. Barker, Melody A. Keena, Michael D. Ormsby, Allan L. Carroll

**Affiliations:** ^1^ Natural Resources Canada, Canadian Forest Service, Laurentian Forestry Service, Québec, QC, Canada; ^2^ Forest Insect Disturbance Ecology Laboratory, Department of Forest & Conservation Sciences, University of British Columbia, Vancouver, BC, Canada; ^3^ Office of the Chief Forester, Ministry of Forests, Victoria, BC, Canada; ^4^ Oregon Integrated Pest Management Center, Oregon State University, Corvallis, OR, United States; ^5^ Department of Horticulture, Oregon State University, Corvallis, OR, United States; ^6^ United States Department of Agriculture-Forest Service, Northern Research Station, Hamden, CT, United States; ^7^ Office of the Chief Biosecurity Officer, Biosecurity New Zealand, Ministry for Primary Industries, Wellington, New Zealand

**Keywords:** forest insects, invasive species, risk assessment, modelling, risk mapping, climate change

Forests around the globe are facing unprecedented threats from biotic and abiotic factors that challenge the overall health of ecosystems and potentially contribute to climate warming through positive feedback to atmospheric CO_2_ (1–4). Presently, some of the greatest challenges pertain to the rapid spread and resulting tree mortality caused by non-native forest insects (5, 6). Increased global trade and more favorable climates due to climate change have facilitated the establishment of non-native forest insects in new areas where conditions were previously unsuitable for their growth and survival (7, 8). The lack of co-evolutionary history between native trees and invasive insects, combined with the absence of natural enemies in the invaded habitats, has allowed aggressive non-native forest insects to rapidly colonize or kill trees and expand their invaded range in a short period of time (9). The invasion and spread of certain non-native forest insects such as the spongy moth [*Lymantria dispar* (L.)], the hemlock woolly adelgid [*Adelges tsugae* (Annand)], and the emerald ash borer (*Agrilus planipennis* Fairmaire) can have both short- and long-term impacts on forest health through the disruption of a range of ecological processes and ecosystem services (5, 9, 10). For example, some non-native forest insects can cause extensive defoliation or tree mortality (5), alter plant community composition (11), change soil hydrology (12), and influence carbon and nutrient cycling (13, 14). The socio-economic costs and human health-related impacts of invasive forest insects can be very significant (15, 16). These impacts will continue to be a concern owing to the consistently large number of non-native insects being intercepted at ports of entry (17–19).

Given the far-reaching consequences of non-native forest insect invasions on the environment and the economy, it is critical to address this threat for the long-term sustainability of urban and natural forest ecosystems. Pest risk models and maps are key tools for assessing the risk posed by such threats as they enable quantification and visualization of the invasion and damage potential of non-native pests (20). They may incorporate transport pathways, known or presumed responses to one or more environmental drivers and/or the dispersal capacity of invasive species to predict their seasonal activity (phenology) and population dynamics or to predict the likelihood of pest introduction, establishment, and spread (21, 22). A wide range of modeling methods are available, such as correlative (e.g., ecological niche models), semi-mechanistic (e.g., CLIMEX), and process-based approaches (e.g., insect phenology models) (20, 22, 23). When integrated with impact assessments, pest risk models can be used for decision support to guide management and surveillance strategies (21). This editorial aims to summarize published articles that cover the above-mentioned aspects under the Research Topic, Forest Insect Invasions – Risk Mapping Approaches and Applications, highlighting the latest work in predictive modeling and pest surveillance.

Maps that predict the phenology of invasive insects can support efforts to detect and control populations because decision-makers often target life stages that are more observable (e.g., larvae vs. adults of wood-boring beetles) or more susceptible to control tactics such as pesticide treatments (24, 25). Similarly, maps that predict the risk of establishment and spread of invasive insects can support surveillance programs by identifying areas that have both suitable environments for population persistence and a high likelihood of pest arrival (26–28). In this Research Topic, Takeuchi et al. presented a web-based spatial analytic framework that produces forecasts of the phenology, climate suitability, and spread of high-priority invasive insects and diseases that threaten forest and agricultural ecosystems in the United States (US). The Spatial Analytic Framework for Advanced Risk Information Systems (SAFARIS) is publicly available and was developed to support the surveillance efforts conducted by the US Department of Agriculture, Animal and Plant Health Inspection Service, Plant Protection and Quarantine (USDA-APHIS-PPQ) program in the continental US. The utility of the SAFARIS system was demonstrated using two invasive insect species that threaten forests in the US, the oak ambrosia beetle (*Platypus quercivorus* Murayama, 1925) and the spongy moth. In a related study, Barker et al. developed and validated a spatial model that combines forecasts of emerald ash borer (EAB) phenology and risk of establishment to help with the development and implementation of effective management strategies against this major invasive pest of ash (*Fraxinus* spp.) in North America and other regions such as Europe. The model for EAB is one of 16 developed for use in the Degree-Days, Risk, and Phenological event mapping (DDRP) platform, which serves as an open-source modeling tool to help detect, monitor, and manage invasive threats (29). Near-real-time model forecasts of EAB for the continental US are available on two websites to support decision-making for the detection of new establishments and for controlling existing populations with pesticide treatments and parasitoid introductions.

Certain invasive insect species can be monitored using natural methods such as assessing the prey catches of predatory insects. This approach is particularly useful when traditional monitoring methods are laborious and expensive, as is the case with EAB. A study by Rutledge and Clark examined EAB catches by a predatory wasp, *Cerceris fumipennis* Say, to describe the occurrence and proportional abundance of EAB among all buprestids caught by *C. fumipennis*. The paper presents ten years of biosurveillance data on EAB in the state of Connecticut, US, to identify the time from first detection to a population decline, which was nine years on average. The outward expansion of EAB from an epicenter assessed through the prey-capture methodology supports findings on EAB dispersal using other frameworks (e.g., tree infestations) (30).

Trotter et al. introduced the Asian Longhorned Beetle Hazard Management and Monitoring (ALBHMM) 2.0 tool, which offers a structured approach to tracking progress toward eradication and optimizing future management efforts for the Asian longhorned beetle [*Anoplophora glabripennis* (Motschulsky)], an invasive wood borer native to China and Southeast Asia that attacks multiple hardwood species (17, 31). The Asian longhorned beetle has been introduced into the US, Canada, and Europe (17–19) raising phytosanitary concerns that have led to the adoption of policies aimed at preventing its spread and eradicating established populations (32). The beetle poses a threat to both urban tree cover and forested landscapes, making eradication efforts crucial. The ALBHMM 2.0 tool integrates information on beetle dispersal, surveys, and management activities (tree removal) to quantify changes in infestation risk at the landscape scale, allowing for measurement of changes in infestation risk over time to monitor eradication progress. The utility of the tool was demonstrated with infestation data from three US states with varying beetle dispersal behaviors and eradication program histories.

In summary, this Research Topic sought to collate contributions describing novel techniques and recent advancements in modeling to assess and reduce the risk posed by non-native forest insect invasions. The studies published here presented innovative tools (SAFARIS, DDRP, and ALBHMM 2.0) to support and improve strategic and tactical decisions for insect surveillance and management, in addition to a novel biosurveillance approach for population monitoring (EAB monitoring using a predatory wasp). The modeling approaches included in this Research Topic provide unique perspectives on pest risk assessment due to differences in their modeling frameworks, but they can also be complementary. For instance, the DDRP system, which was used to model EAB in Barker et al., is complementary to SAFARIS in that model forecasts can support decision-making for the surveillance of invasive pests and for the management of already-established invasive pests.

The adoption of pest forecasting tools and map products requires engagement with end users such as pest control managers, government officials, and the general public (33). However, model complexity, a lack of training opportunities for end users, and insufficient outreach can hinder the broader uptake of these tools and products. Therefore, providing educational opportunities, requesting user feedback, and improving the delivery and formats of map products based on the feedback received are recommended (33). Considering the potential for future invasions of destructive forest insects under climate change, utilizing new and powerful technologies to model pest risk and filling gaps in end-user outreach and training are critical to safeguarding forest health.

## Author contributions

KS: Writing – review & editing, Writing – original draft. VS: Writing – review & editing, Writing – original draft. BB: Writing – review & editing, Writing – original draft. MK: Writing – review & editing. MO: Writing – review & editing. AC: Writing – review & editing.
